# Core Fucosylation of the T Cell Receptor Is Required for T Cell Activation

**DOI:** 10.3389/fimmu.2018.00078

**Published:** 2018-01-29

**Authors:** Wei Liang, Shanshan Mao, Shijie Sun, Ming Li, Zhi Li, Rui Yu, Tonghui Ma, Jianguo Gu, Jianing Zhang, Naoyuki Taniguchi, Wenzhe Li

**Affiliations:** ^1^College of Basic Medical Sciences, Dalian Medical University, Dalian, China; ^2^Clinical Laboratory, Dalian Municipal Central Hospital, Dalian, China; ^3^Division of Regulatory Glycobiology, Institute of Molecular Biomembrane and Glycobiology, Tohoku Medical and Pharmaceutical University, Sendai, Japan; ^4^School of Life Science and Medicine, Dalian University of Technology, Panjin, China; ^5^Systems Glycobiology Research Group, Advanced Science Institute, RIKEN, Saitama, Japan

**Keywords:** core fucosylation, T cell receptor, T cell activation, systemic lupus erythematosus, T–B cell interaction

## Abstract

CD4^+^ T cell activation promotes the pathogenic process of systemic lupus erythematosus (SLE). T cell receptor (TCR) complex are highly core fucosylated glycoproteins, which play important roles in T cell activation. In this study, we found that the core fucosylation of CD4^+^ T cells was significantly increased in SLE patients. Loss of core fucosyltransferase (Fut8), the sole enzyme for catalyzing the core fucosylation of N-glycan, significantly reduced CD4^+^ T cell activation and ameliorated the experimental autoimmune encephalomyelitis-induced syndrome in Fut8^−/−^ mice. T cell activation with OVA_323–339_ loaded major histocompatibility complex II (pMHC-II) on B cell was dramatically attenuated in Fut8^−/−^OT-II CD4^+^ T cells compared with Fut8^+/+^OT-II CD4^+^ T cells. Moreover, the phosphorylation of ZAP-70 was significantly reduced in Fut8^+/+^OT-II CD4^+^ T cells by the treatment of fucosidase. Our results suggest that core fucosylation is required for efficient TCR–pMHC-II contacts in CD4^+^ T cell activation, and hyper core fucosylation may serve as a potential novel biomarker in the sera from SLE patients.

## Introduction

Systemic lupus erythematosus (SLE) is a severe autoimmune disease that characterized by the production of autoantibodies and the subsequent inflammatory disorders (1). Although the pathogenesis is not completely understood, the activation of CD4^+^ T cells seems plays an essential role in the onset and development of SLE (2). Appropriate CD4^+^ T cell activation is crucially important for adaptive immune responses and autoimmunity, but hyper-activation of these cells results in autoimmune diseases. T cell recognition of peptide-loaded major histocompatibility complex II (pMHC-II) on the antigen-presenting cells (APCs) by T cell receptors (TCRs) is the most important checkpoint for CD4^+^ T cell activation (3). During antigen recognition, the CD4 coreceptor binds to the non-polymorphic surfaces of the membrane-proximal domains of the same pMHCs, which results in a marked increase in the sensitivity of T cells to pMHCs on APC. When adequate agonistic TCR signaling creates a favorable microenvironment for binding, CD28-B7 molecules provide costimulatory lower the thresholds for TCRs triggering and activation. The signaling through TCR induces a conformational change in leukocyte function-associated antigen-1, which greatly increases its affinity for intercellular adhesion molecule 1 and contributes to immune synapse formation T cells and APC (4, 5).

Glycosylation plays a regulatory and often pivotal role in T cell activation (6–10). Several studies have reported that glycosylation could contribute to higher activation thresholds of T cells. For instance, β1,6*N*-acetylglucosaminyltransferase V (Mgat5) deficiency mediates lower T lymphocyte activation thresholds, and subsequently improves T cell activity *in vitro* and results in autoimmune disease *in vivo* (11, 12). Deletion of sialyltransferase ST3Gal-1 increase the sensitivity of TCRs to low-affinity ligands in CD8^+^ T cells (13). Fucosyltransferase 1 transgenic mice show increased TCR signaling and apoptosis that results in thymocyte maturation arrest (14). Notably, reduced N-glycosylation of TCR chains can improve functional avidity and recognition by T cells (15) suggesting that the glycosylation of TCR has a unique role in the regulation of T cell activation.

T cell receptors are heavily core-fucosylated glycoproteins. The core fucosylation of protein is catalyzed by core fucosyltransferase (Fut8), which transfers fucose residue from GDP-fucose to the innermost *N*-acetylglucosamine (GlcNAc) residue of N-linked glycans *via* an α1,6 linkage in the Golgi apparatus of mammalians (Figure S1 in Supplementary Material). Fut8-mediated core fucosylation is an important post-translational process (16), which regulates protein conformation, stability, and functional expression. Studies have shown that the N-glycans at Asn^70^ [GlcNAc(α1,6Fuc)-β1,4GlcNAc: (A2G2F)], Asn^185^ (A2G2F), and Asn^203^ in the α chain (Cα) and Asn^236^ in the β chain (Cβ) extend from the surface of TCR on *Drosophila melanogaster* cells (6, 17). Interestingly, they found that the Cα and Cβ of TCR were connected by the hydrogen bonds of the core fucose residue from Asn^185α^ (A2G2F) to side chains of Glu^181β^ and Ser^182β^ (6, 17), suggesting a crucial role of core fucosylation on the conformation of TCR. However, to the best of our knowledge, none of the previous studies had addressed the regulatory role of TCR core fucosylation on CD4^+^ T cell activation.

B cells play a role in evoking T cell responses by functioning as APCs, and the presentation of peptide by MHC-II on the B cells initiates T cell activation (18). Therefore, it is reasonable to anticipate that the core fucosylation has significant functional implications in T–B cell interaction, and thus affect the CD4^+^ T cell activation. In this study, we provide the first confirmation that SLE patients exhibited hyper core fucosylation on CD4^+^ T cells, which significantly enhanced the activation of their CD4^+^ T cells. Knockout of Fut8 gene resulted in attenuated T–B cell interaction *via* TCR–pMHC and the consequential reduced CD4^+^ T cell activation. Our data suggest that the core fucosylation may serve as a potential novel biomarker with promising clinical and therapeutic implications in SLE patients.

## Materials and Methods

### Mice

Fut8^−/−^ mice were generated as previously described (19), and homozygous wild-type (Fut8^+/+^) and Fut8^−/−^ mice on the C57BL/6 background were obtained by crossing heterozygous Fut8^+/−^ mice (C57BL/6). OT-II (Jackson Laboratory) is a C57BL/6 TCR transgenic strain, expressing a receptor specific for peptide OVA_323–339_. Fut8^+/+^OT-II mice and Fut8^−/−^OT-II mice were generated by crossing heterozygous Fut8^+/−^OT-II mice. Mice were maintained in the specific pathogen-free laboratory animal facility of Dalian Medical University. All animal work was approved by the Ethics Committee at the Dalian Medical University.

### Patients

Serum samples were collected from a total of 17 SLE patients (14 women, 3 men; mean age, 49 years; range, 18–67 years) with and healthy controls (12 women, 12 men; mean age, 18–48 years) (Table S1 in Supplementary Material). The diagnosis of underlying disease was made based on clinical manifestation, serology, imaging, and/or histopathology. These participants were Chinese, recruited at Dalian municipal central hospital. The anti-nuclear antibodies (ANA) titers of AD patients were detected with using Anti-nuclear Antibodies IgG Kit (EUROIMMUN, Germany). The Ethics Committee at the hospital approved the study protocol.

### Antibodies

Anti-CD16/32 (2.4G2), anti-CD3(145-2c11), anti-CD28 (37.51), FITC-anti-MHC II (M5/114.15.2), FITC-anti-CD69 (H1.2F3), PE-labeled anti-CD4 (GK1.5), APC-labeled anti-CD8 (53-6.7), biotin-labeled anti-TCRβ (H57-597), and PE-Cy5-labeled anti-TCRβ (H57-597) were obtained from e-Bioscience; anti-GAPDH, horseradish peroxidase (HRP)-conjugated goat anti-mouse IgG and HRP-conjugated donkey anti-human IgG were obtained from proteintech; additional biotin-conjugated lens culinaris agglutinin (LCA) were purchased from Vector; anti-TCRαβ (ab25336), anti-pZAP70 (ab194800), anti-ZAP70 (ab32410), Natural streptavidin protein (FITC) (ab136201), and streptavidin (HRP) (ab7403) were purchased from Abcam.

### Histological Analysis

Formalin-fixed tissue samples were paraffin-embedded and sections were analyzed by hematoxylin–eosin (H&E) staining. The sections were stained with biotin-conjugated LCA. Briefly, sections were deparaffinized three times in xylene and hydrated through a 100, 90, 80, and 70% ethanol to phosphate-buffered saline (PBS). To quench the endogenous peroxidase activity, slides were incubated with 3% H_2_O_2_ for 30 min. Then, the slides were incubated with biotin-conjugated LCA, and washed three times with PBS. The slides probed with HRP–streptavidin for 30 min, and visualized with 3,3′-diaminobenzidine. The intensity of LCA-positive staining in the spleen was analyzed by integrated optical density using Image-Pro^®^ Plus software (version 6.0; Media Cybernetics, USA).

### Cell Lysate

Cells were solubilized in lysis buffer [Tris–HCl (50 mM), 1% Triton X-100, 10% glycerol, phenylmethylsulfonylfluoride (100 µM), leupeptin (5 µg/mL), aprotinin (1 µg/mL), NaF (100 mM), 150 mM NaCl, 2 mM EDTA, and sodium orthovanadate (1 mM)] for 15 min at 4°C. Cell lysate was centrifuged at 20,000 × *g* for 10 min at 4°C, and the supernatant was subjected to immunoprecipitation or Western blot, as indicated below.

### Fut8 Enzyme Activity Assay

The Fut8 enzyme activity was measured by using the previous method (20). Five micrograms cell lysates as the enzyme source were added to the assay buffer (200 mM MES, 1% Triton X-100) supplemented with donor (500 µM GDP-L-fucose) and substrate [50 µM GnGn-Asn-4-(2-pyridylamine) butylamine (PABA)]. The mixture was incubated at 37°C for 8 h, and the reaction was stopped by heating at 100°C for 5 min. The reactive solution was then centrifuged at 12,000 × *g* for 10 min, and 10 µL of the reaction products were subjected to high-performance liquid chromatography (HPLC) with a Fluorescent detector (Waters Corporation, USA). The excitation and emission wavelengths are 320 and 400 nm, respectively.

### PCR Array

Total RNAs were extracted from Fut8^+/+^ SPLs and Fut8^−/−^ SPLs with TRIzol reagent (Takara Bio). Mouse T Cell and B Cell Activation PCR Array (SA Biosciences) was carried out according to the protocol of the manufacturer. The difference of gene expression between Fut8^+/+^ SPLs and Fut8^−/−^ SPLs was calculated.

### Animal Immunization

Mouse was immunized by subcutaneous injection with 200 µg OVA mixed with an equal volume of complete Freund’s adjuvant (CFA) (Sigma). Two weeks later, mice were immunized with 200 µg OVA by subcutaneous injection. Mice sera were collected at 0, 7, and 14 days post-immunization.

### Enzyme-Linked Immunosorbent Assay (ELISA)

The concentrations of IL-2 analyzed using mouse IL-2 ELISA kits (Boster Biological Engineering, Wuhan, China), according to the manufacturer’s instructions. The concentrations of IL-2 were calculated according to a standard curve prepared using samples of known concentration. The absorbance was measured at a test wavelength of 450 nm with a microplate reader.

The immunoglobulin isotypes were measured by mouse mAb isotyping reagents (Sigma).

### Cell Proliferation Assay

The growth rate of cells was measured using MTT assay. CD4^+^ T cells (1 × 10^6^) were cultured in 96-well culture plate with anti-CD3ε (2 µg/mL) and anti-CD28 mAbs (1 µg/mL). After 48 h of incubation, each well was added 10 µL of MTT solution, and then the absorbance was analyzed by a microplate reader (Thermo Multiskan Ascent, Finland) at 570 nm.

In addition, T cell proliferation with the OVA_323–339_-loaded B cells was analyzed by carboxyfluorescein diacetatesuccinimidyl ester (CFSE, Sigma) dilution methods. CD4^+^ T cells (1 × 10^6^) were purified, and then labeled with 5 µM CFSE in PBS for 8 min at room temperature and coincubate with OVA_323–339_ loaded Fut8^+/+^ OT-II B cells (1 × 10^6^) for 48 h, and then analyzed by flow cytometric analysis.

### Western Blot and Lectin Blot Analysis

Protein samples were electrophoresed on 10% polyacrylamide gels. After electrophoresis at 240 mA for 30 min, proteins were transferred to PVDF membranes. Membranes were blocked in 5% BSA in TBS-T (10 mM Tris–HCl, 150 mM NaCl, and 0.1% Tween 20) at room temperature for 1 h, and then incubated with the biotin-labeled LCA, which preferentially recognizes the core fucose, or primary Abs in 1% BSA in TBS-T overnight at 4°C. After washing, the membranes were covered with the HRP-conjugated streptavidin or HRP-labeled secondary Abs at room temperature for 1 h, and visualized with an ECL system (Amersham).

### Immunoprecipitation

Cell extracts (500 µg) were mixed with 20 µL of Protein G-Sepharose (50%) and corresponding Abs, and then incubated at 4°C overnight with continuous rotation. After washing three times in lysis buffer, the pull downed samples were boiled for 5 min in Laemmli sample buffer with or without 2-mercaptoethanol.

### T–B Cell Conjugate Formation

Conjugate formation between T cells and B cells were carried out as described previously with slight modification (21). T cells and 1 µg/mL OVA_323–339_ (NH_2_-ISQAVHAAHAEINEAGR-COOH)-pulsed B cells were mixed at a 1:1 ratio and a quick centrifugation to initiate cell–cell contact. To observe conjugate formation, B cells and T cells were labeled with MHC-II-FITC and TCRβ-PE-Cy5 before mixing. T–B cell conjugates were then analyzed by flow cytometry to determine the percentage of T–B cells that had both TCRβ-PE-Cy5 and MHC-II-FITC positive staining.

### Remove of Core Fucose on Surface of T Cells

Purified CD4^+^ T cells (4 × 10^6^) were treated with 100 mU Glyko^®^ α(1-2,3,4,6) Bovine Kidney Fucosidase (GKX-5006, Prozyme), incubate 3 h at 37°C in the reaction buffer. The enzyme reaction was terminated by centrifuging at 2,500 × *g* for 5 min and the cells were collected.

### Confocal Microscopy

Conjugate formation between T cells and B cells were carried out as described previously with slight modification (21). T cells and 1 µg/mL OVA_323–339_ pulsed B cells were mixed at a 1:1 ratio and a quick centrifugation to initiate cell–cell contact. Cell–cell conjugates were subsequently transferred to poly-d-lysine coated coverslips and incubated at 37°C for 30 min. Cells were fixed with 4% PFA for 20 min, and then blocked with 5% BSA and anti-CD16/CD32 (2.4G2) mAb for 30 min. After washing, cells were stained with anti-MHC-II Ab for 1 h. All images were taken using a spinning disk confocal microscope (Leica).

### MACS Magnetic Cell Sorting

Single splenic cell suspensions were prepared by first grinding the tissues and then by passage through 30-µm nylon mesh. Red blood cells were lysed by incubation with 0.14 M NH4Cl and 20 mM Tris (pH7.4) for 3 min at room temperature. After the lysis of red blood cells, CD4^+^ T cells and B cells were positively isolated with anti-CD4 Ab and anti-CD45R Ab-conjugated magnetic beads (Miltenyi Biotec). The purified cell populations were detected by fluorescence activated cell sorting analysis.

### Flow Cytometric Analysis (FACS)

Cells were isolated from tissue and incubated with an anti-CD16/CD32 (2.4G2) mAb to block Fcγ receptors. The cells were stained on ice for 15 min with several combinations of mAbs, as indicated in the figure legends. Flow cytometry was performed on a FACS-Calibur (Becton Dickinson, Mountain View, CA, USA) and analyzed using FlowJo software (Tree Star).

### Induction of Experimental Autoimmune Encephalomyelitis (EAE)

For EAE induction, Fut8^+/+^ and Fut8^−/−^ mice were subcutaneously injected with 100 µg of myelin oligodendrocyte glycoprotein peptides (MOG_35–55_) emulsified in CFA. Then, mice were injected intraperitoneally with 200 µg pertussis toxin (PTX) (List Biological Laboratories) on 0, 1, and 2 days. Clinical assessment of EAE was performed according to the following scale: 0, no disease; 1, limp tail; 2, hind-limb weakness; 3, partial hind-limb paralysis; 4, complete paralysis of hind-limbs; and 5, moribund state.

### Statistical Analysis

Student’s *t*-test was used for statistical analysis. Data are presented as mean values ± SEM, or as mean values ± SD. A probability value of *p* < 0.05 was considered significant. *p < 0.05, **p < 0.01, ***p < 0.001.

## Results

### Core Fucosylation Is Significantly Upregulated in the Sera and CD4^+^ T Cells of SLE Patients

**Figure 1:**
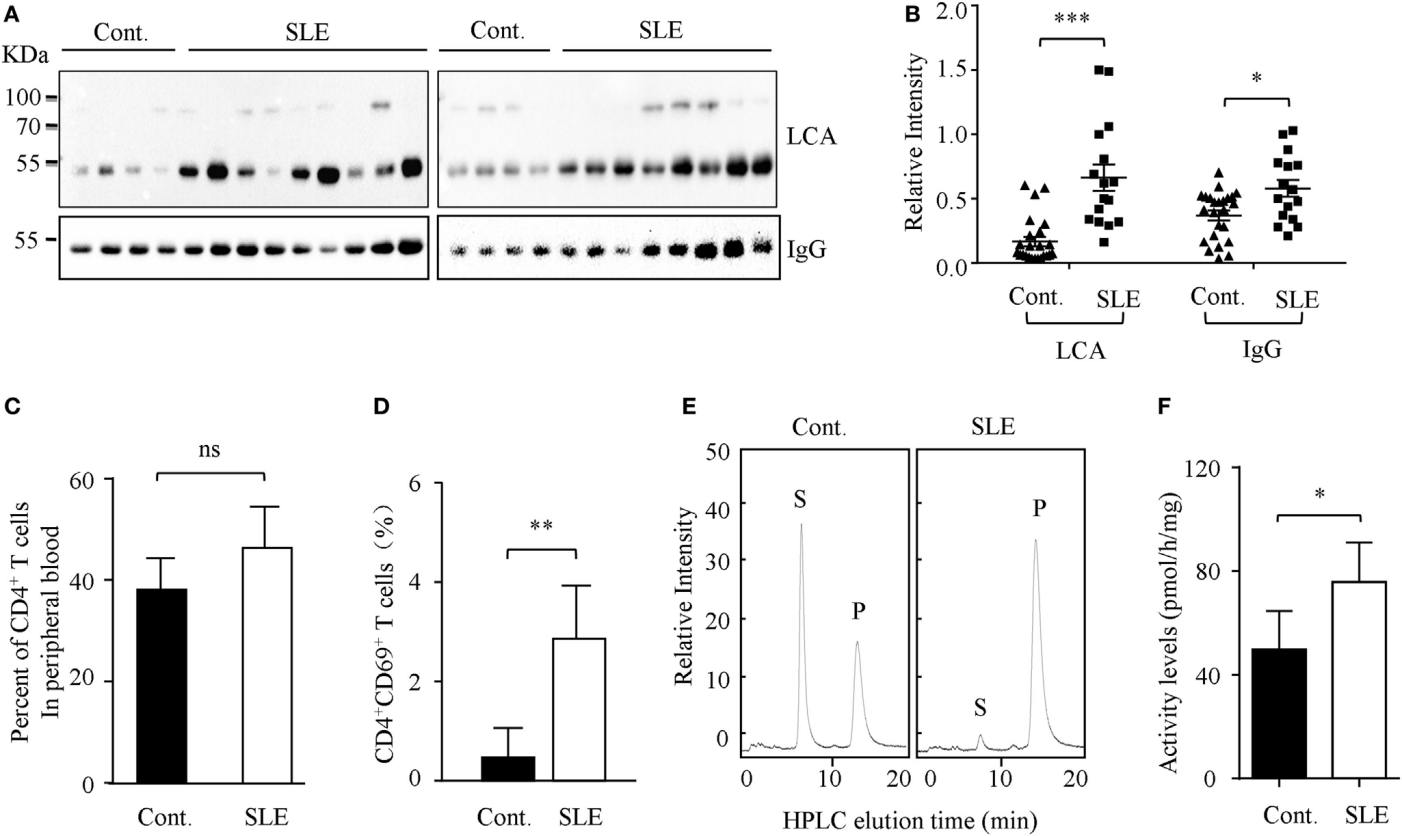
Core fucosylation was significantly increased in the systemic lupus erythematosus (SLE) patients. **(A)** The sera of SLE patients were analyzed by lens culinaris agglutinin (LCA) blot and Western blot. Plates were incubated with biotin-conjugated LCA (1:20,000). Comparable results were obtained in three independent experiments. **(B)** Densitometric analysis of the bands of IgG and LCA in sera in SLE patients. Data are shown as mean values ± SEM (*p < 0.05; ***p < 0.001). **(C)** The percentage of CD4^+^ T cells in the peripheral blood of SLE patients. **(D)** The percentage of CD4^+^CD69^+^ T cells in the peripheral blood of SLE patients (*n* = 17). **(E,F)** FUT8 activities in the CD4^+^ T cells of SLE patients by high-performance liquid chromatography (HPLC). CD4^+^ T cells were isolated with anti-CD4 Ab-conjugated magnetic beads. The sorted cell populations were routinely more than 96% pure. Five micrograms cell lysates as the enzyme source were mixed with the assay buffer. After incubation at 37°C for 8 h, 10 µL of the supernatant was subjected to HPLC. Activity was expressed as pmol of GDP-fucose transferred to the acceptor per hour per milligram of protein. Data are shown as mean values ± SD (*n* = 17; ns, not significant; *p < 0.05; **p < 0.01). S is the peptide substrate and P is the product of fucosylation.

Higher circulating levels of ANA were detected in sera from SLE patients. In this study, we found that core fucosylation was dramatically increased in the sera from patients with SLE, as evidenced by LCA, which preferentially recognizes the core fucose structure (22) (*p* < 0.001) (Figures 1A,B). The expression of IgGs was also upregulated in sera of the SLE patients (*p* < 0.05) (Figure 1B). These observations pinpoint the contribution of hyper core fucosylation to SLE severity and pathogenesis.

The hyperactivity of B cells in SLE is T cell dependent, and CD4^+^ T cell activation plays a crucial role in SLE pathogenesis (23). We found that the percentage of CD4^+^ T cells in the peripheral blood of SLE patients is similar to those of healthy control (Figure 1C). However, the percentage of CD4^+^CD69^+^ T cells was significantly increased in the SLE (*n* = 17) (Figure 1D). Moreover, the enzyme activity of Fut8 was dramatically increased in the CD4^+^ T cells isolated from the SLE patients (Figures 1E,F), indicated that increased core fucosylation in SLE patients correlates with CD4^+^ T cell activation.

### Lack of Core Fucosylation Ameliorated EAE Symptoms with Reduced CD4^+^ T Cell Activation

**Figure 2:**
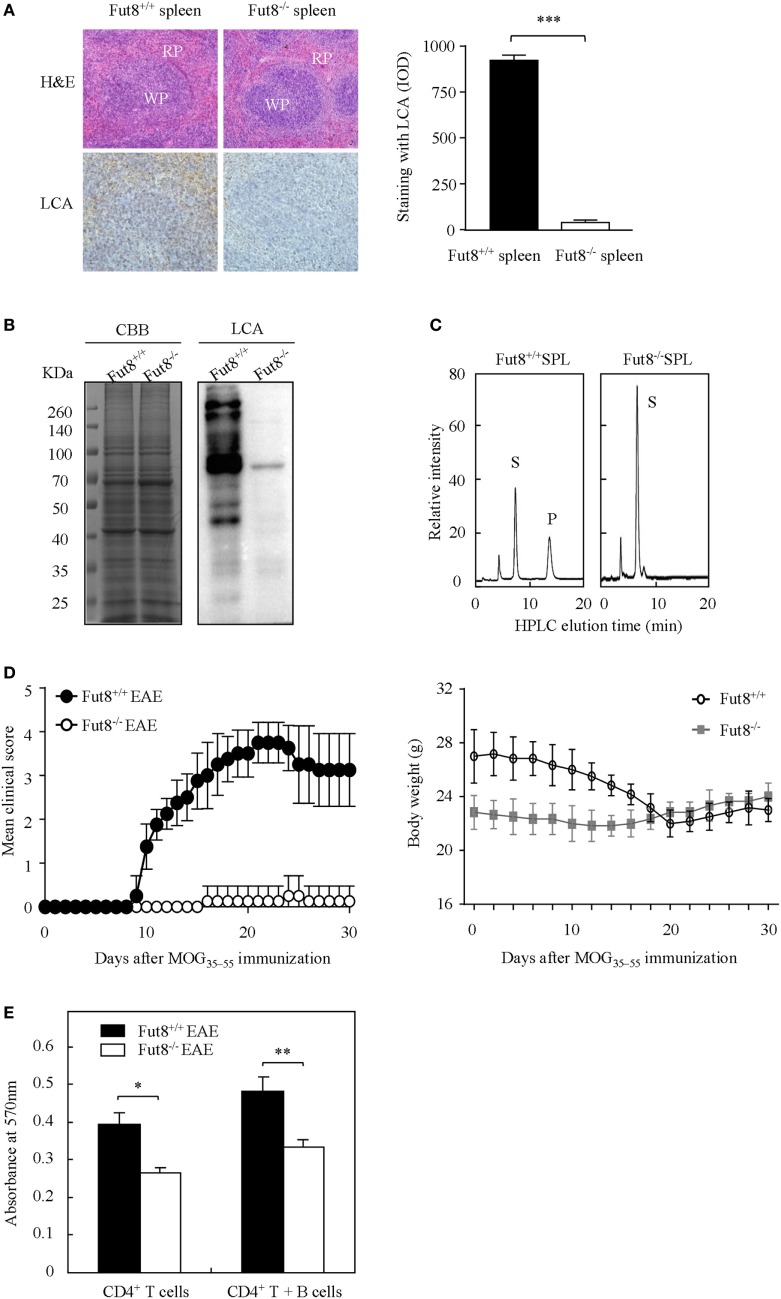
The experimental autoimmune encephalomyelitis (EAE) model was slightly induced in Fut8^−/−^ mice. **(A)** Immunohistochemical analysis of Fut8^+/+^ spleen and Fut8^−/−^ spleen. The paraffin sections of spleens were deparaffinized and hydrated through a graded series of ethanol to phosphate-buffered saline. One section was assessed by hematoxylin–eosin staining (magnification ×200). RP, red pulp; WP, white pulp. Another section was incubated with biotin-conjugated lens culinaris agglutinin (LCA) (1:200) for 1 h. Finally, the slides were visualized with 3,3′-diaminobenzidine. The staining with LCA was showed by integrated optical density analysis. Data are shown as mean values ± SD (***p < 0.001). **(B)** Lectin bolt of Fut8^+/+^ SPL and Fut8^−/−^ SPL. The SPL lysates were run on 10% SDS-PAGE gel and stained with Coomassie blue staining and LCA (1:5,000). **(C)** High-performance liquid chromatography (HPLC) analysis of Fut8 activity. Fut8 activities were examined using fluorescence-labeled sugar chain, GnGn-Asn-PABA, as an acceptor substrate, as described in Section “Materials and Methods.” The substrate (S) and Fut8 product (P) were eluted at 8 and 15 min, respectively. **(D)** Disease score of mice in Fut8^+/+^ and Fut8^−/−^ mice EAE model. EAE induction of Fut8^+/+^ and Fut8^−/−^ mice (*n* = 7). Mice were immunized with 100 µg MOG_35–55_ peptide in complete Freund’s adjuvant and injected with 200 µg pertussis toxin, and detected the signs of EAE daily for 30 days. Comparable results were obtained in four independent experiments. Body weights of mice were measured every 2 days after EAE induction of Fut8^+/+^ and Fut8^−/−^ mice (*n* = 7). **(E)** Cell proliferation from EAE mice. Purified splenic CD4^+^ T cells and/or MOG_35–55_ peptide-loaded B cells from EAE models were incubated for 48 h at 37°C, and then the cell proliferation was detected by MTT assay. Comparable results were obtained in four independent experiments. Data are shown as mean values ± SD (*p < 0.05; **p < 0.01).

Wild-type (Fut8^+/+^) and Fut8^−/−^ mice were generated previously (19). Histological analyses of the splenic architecture of Fut8^+/+^ and Fut8^−/−^ mice were unremarkable in H&E staining. The Fut8 products, core-fucosylated N-glycans, are ubiquitously expressed in the Fut8^+/+^ spleen, as confirmed by LCA (Figure 2A), while those were abolished in Fut8^−/−^ spleens. LCA blot analysis also confirmed the knockout of Fut8 expression in the whole cell lysates of Fut8^−/−^ SPLs (Figure 2B). The FUT8 enzymatic activity was not detected in the Fut8^−/−^ SPLs using HPLC analysis (Figure 2C).

Experimental autoimmune encephalomyelitis is an activated CD4^+^ T cell-mediated autoimmune disease model. Peptides (MOG_35–55_) and PTX could induce the migration of activated T cells through the blood–brain barrier and caused several neurologic symptoms. To determine the association between core fucosylation and the activation of CD4^+^ T cells, EAE models were established using Fut8^+/+^ and Fut8^−/−^ mice. EAE is actively induced but appear more quickly upon adoptive transfer of activated MOG_35–55_-specific T cells in Fut8^+/+^ mice (Figure 2D; Video S1 in Supplementary Material), while Fut8^−/−^ mice showed slight EAE symptoms (Figure 2D; Video S2 in Supplementary Material). The body weights of Fut8^+/+^ mice were significantly reduced, but no change was found in Fut8^−/−^ mice during EAE induction (Figure 2D). In addition, the proliferation of CD4^+^ T cells was significantly decreased in Fut8^−/−^ EAE mice. Moreover, the proliferation of CD4^+^ T cells with MOG_35–55_-loaded B cells was remarkably reduced by de-core fucosylation (Figure 2E).

### Lack of Core Fucosylation Suppressed the IgG Class-Switching by Impaired CD4^+^ T Cell Activation

**Figure 3:**
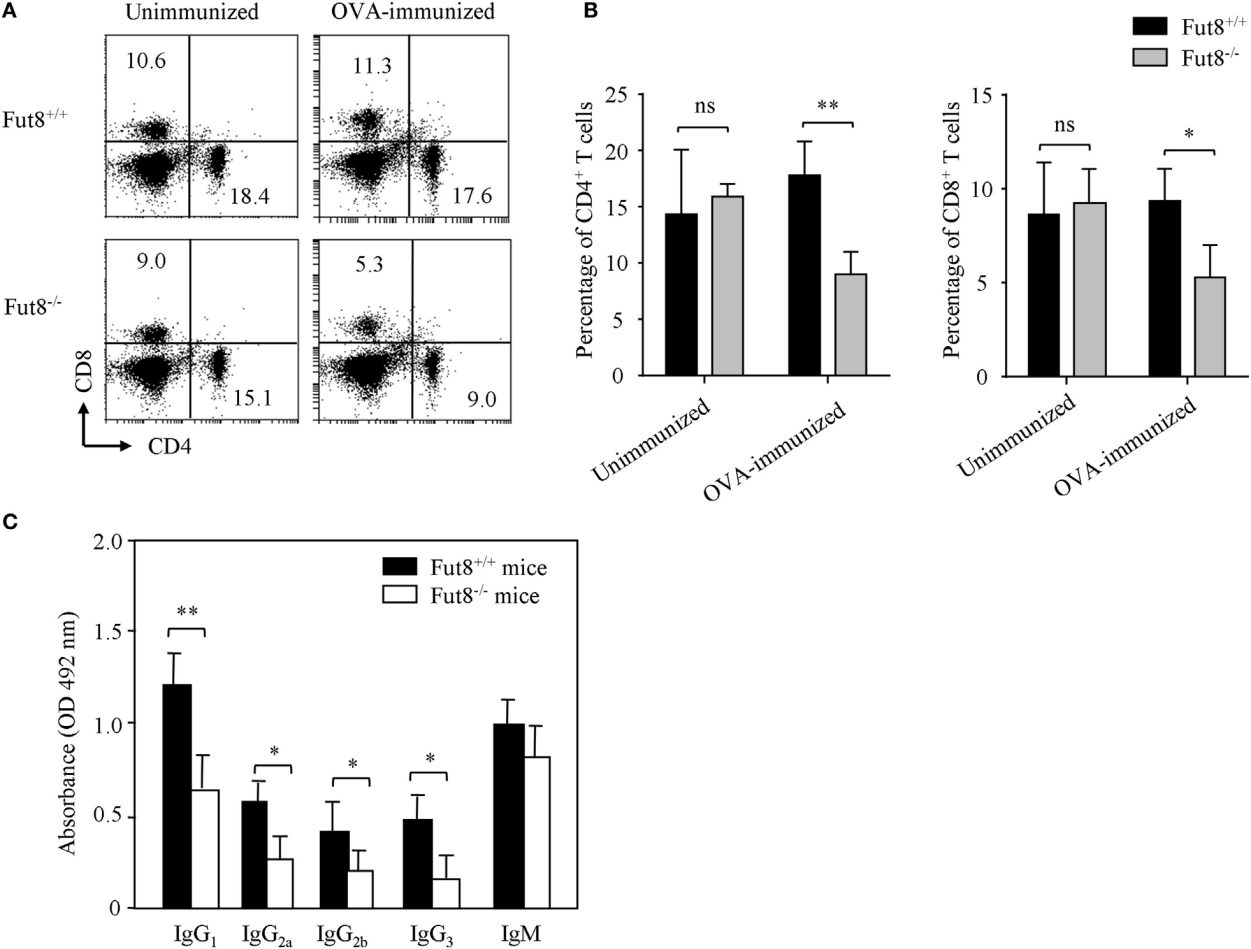
Lack of core fucosylation suppressed the IgG class-switching by impaired CD4^+^ T cell activation. **(A,B)** Flow cytometry analysis of the proportion of CD4^+^ and CD8^+^ cells. SPLs were obtained from Fut8^+/+^ and Fut8^−/−^ mice with or without OVA immunization. SPLs were filtered through nylon mesh, and resuspended. Numbers indicate the percentage of the total spleen cells within this quadrant, and 10,000 events were acquired for each analysis. Data are representative of three independent experiments. Data are shown as mean values ± SD (ns, not significant; *p < 0.05; **p < 0.01). The number of Fut8^+/+^CD4^+^ T and Fut8^−/−^CD4^+^ T cells were (1.29 ± 0.32) × 10^7^ and (6.38 ± 0.89) × 10^6^ of the SPLs, respectively (*n* = 5). **(C)** Comparison of the levels of serum immunoglobulin isotypes in Fut8^+/+^ and Fut8^−/−^ mice after OVA immunization. The immunoglobulin isotypes are compared by mouse mAb isotyping reagents (Sigma). Comparable results were obtained in three independent experiments. Data are shown as mean values ± SD (*p < 0.05; **p < 0.01).

Flow cytometry analysis revealed that, although Fut8^−/−^ mice contained normal proportions of CD4^+^ and CD8^+^ T cell populations in the spleen (Figures 3A,B), they were markedly reduced after OVA immunization contrast with the Fut8^+/+^ mice (Figures 3A,B). Immunoglobulin class-switching is a biological mechanism that changes a mature B cell’s production of antibody *via* its B cell receptor from one class to another. For example, from an isotype called IgM to an isotype called IgGs. To illustrate the effects of Fut8 in the class-switching of immunoglobulin, we measured the class-switched (IgGs of different subclasses) and non-switched (IgM) in the sera of Fut8^+/+^ and Fut8^−/−^ mice using mouse mAb isotyping reagents. In 4-week-old Fut8^−/−^ mice, the amounts of IgG_1_, IgG_2a_, IgG_2b_, and IgG_3_ were significantly lower than those in Fut8^+/+^ mice after OVA immunization, while those of IgM were relatively normal (Figure 3C). The cytokines, such as IL-4, IL-5, IL-6, and IFNγ secreted by CD4^+^ T cells, contribute to the different IgG class-switching in the mice and human. It is reasonable to consider that the reduced IgG class-switching attributed to the low levels of cytokines secreted by CD4^+^ T cells in Fut8^−/−^ mice (Table 1). In addition, since the TGF receptor signaling was attenuated in the Fut8^−/−^ mice (19), the IgG_3_ class-switching regulated by TGF signaling was also suppressed in the Fut8^−/−^ mice.

**Table 1:** Gene expression of Fut8^−/−^ SPLs after OVA immunization.

Gene name	Gene access number	Fold change (Fut8^+/+^/Fut8^−/−^)
**T-cell activation**		
CD3e	NM-007648	2.15
CD4	NM-013488	3.16
CD8	NM-009858	2.51
Cd40L	NM-011616	2.17
IL-2Rα	NM-008367	3.04
IL-4	NM-021283	2.18
IL-6	NM-031168	2.14
IL-10	NM-010548	2.25
IL-12	NM-008352	3.89
IFNγ	NM-008337	3.08
CXCR4	NM-009911	2.04
CXCL12	NM-001012477	2.07
**B-cell activation**		
CD79a	NM-007655	3.35
CD81	NM-133655	2.82
**Cell signaling**		
MAPKKK	NM_009316	2.41
Vav1	NM-011691	3.28
PIK3	NM-001077495	2.36
PKC	NM-008859	2.00
Cyclin D3	NM-001081636	3.84

### Loss of Core Fucosylation Impaired the Signal Transduction *via* TCR

**Figure 4:**
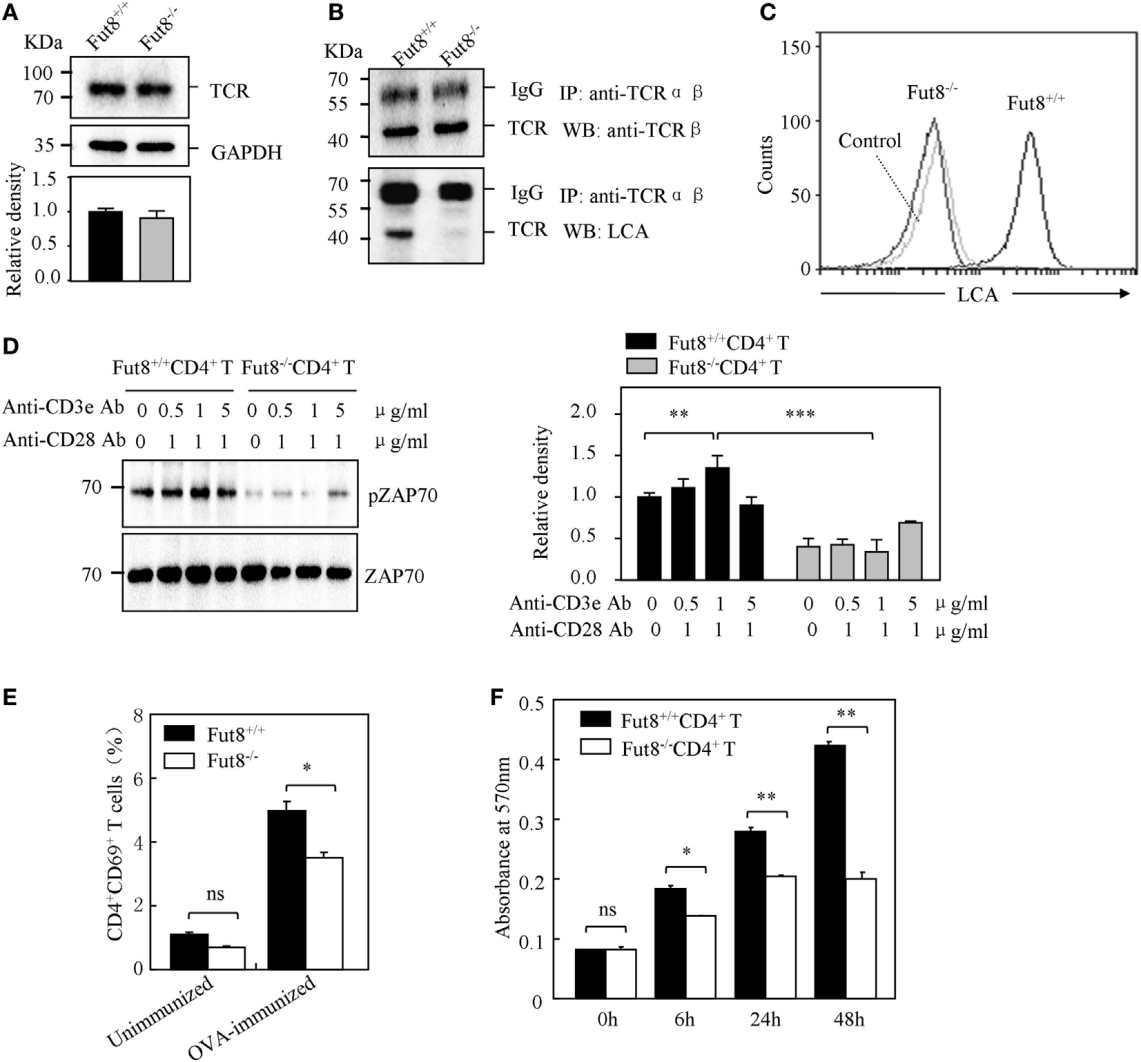
Core fucosylation is required for the activation of CD4^+^ T cells. **(A)** Western blots analysis of T cell receptor (TCR). Whole cell lysates were resolved by SDS-PAGE on a 8% gel, transferred to a PVDF membrane, and probed with anti-TCRβ Ab. Densitometric analysis of the bands of TCRβ normalized against GAPDH. **(B)** Core fucose of N-glycan on TCRβ in Fut8^−/−^CD4^+^ T cells was detected by LCA blot. Whole cell lysates were immunoprecipitated with an anti-TCRαβ antibody. The immunoprecipitates were resolved by SDS-PAGE on a 8% gel, and probed with the LCA and anti-TCRβ Ab. **(C)** Histograms of binding capacity with LCA. Core fucosylation level on the surface proteins of Fut8^+/+^CD4^+^ T and Fut8^−/−^CD4^+^ T cells investigated by FACS analysis. **(D)** Downregulation of phosphorylated Zap70 in Fut8^−/−^CD4^+^ T cells. Purified CD4^+^ T cells were serum-starved and were stimulated with anti-CD3/CD28 Abs for 5 min at 37°C. Cells were lysated in lysis buffer for 15 min on ice. Whole cell lysates were subjected to 10% SDS-PAGE. The blots were probed by anti-pZAP70 Ab and anti-ZAP70 Ab. Densitometric analysis of the bands of pZAP70 normalized against ZAP70. Data are reported as the mean ± SD from three independent experiments (**p < 0.01; ***p < 0.001). **(E)** Loss of Fut8 reduced the CD4^+^CD69^+^ cells populations in the SPL after OVA immunization. SPLs were isolated from OVA-immunized and unimmunized mice (*n* = 5). Cells were stained with anti-CD69 and anti-CD4 Abs, and then detected by FACS. Data are reported as the mean ± SD from three independent experiments (*p < 0.05; ns, not significant). **(F)** Loss of Fut8 decreased the proliferation of CD4^+^ T cells. Purified CD4^+^ T cells were stimulated with anti-CD3ε Ab-coated microbeads and anti-CD28 Ab for 0, 6, 24, and 48 h at 37°C. The growth rates of CD4^+^ T cell were detected by MTT assay. Data are reported as the mean ± SD from three replicate cultures (*p < 0.05; **p < 0.01). The absorbance related to the formazan dye level was measured with a microplate reader at 570 nm.

T cell receptor signaling is very important for T cell activation. As illustrated in Figure 4A, no different expression of TCR was found in the Fut8^+/+^CD4^+^ T and Fut8^−/−^CD4^+^ T cells. Nonetheless, the core fucose of the N-glycans in the molecules was eliminated by a disruption of the Fut8 (Figure 4B). Moreover, the MS spectra of N-glycans released from TCRs were analyzed. It is notable that the high levels of signals were corresponded to the core-fucosylated glycans bearing non-, mono-, or di-galactose in the Fut8^+/+^CD4^+^ T cells, while those were completely disappeared in Fut8^−/−^CD4^+^ T cells (Figure S2 in Supplementary Material). These results further confirmed in mice that TCRs are highly core-fucosylated proteins and contributes to its activities.

In order to examine the role of core fucosylation in the activation of CD4^+^ T cells, we isolated CD4^+^ T cells from Fut8^+/+^ and Fut8^−/−^ mice, and checked the level of core fucosylation. FACS analysis showed that core fucosylation on the cellular surfaces was abolished in Fut8^−/−^ CD4^+^ T cells (Figure 4C). We compared the phosphorylation levels of Fut8^+/+^CD4^+^ T cells with Fut8^−/−^CD4^+^ T cells in response to costimulations with anti-CD3/CD28 Abs. In those comparisons, the levels of pZAP-70 in Fut8^−/−^CD4^+^ T cells were significantly lower than those in Fut8^+/+^CD4^+^ T cells (Figure 4D). Moreover, the populations of CD69^+^ cells (activated T cells) in Fut8^−/−^CD4^+^ T cells were lower than those in Fut8^+/+^CD4^+^ T cells following OVA immunization, while these were similar before immunization (Figure 4E). Furthermore, cell proliferation of Fut8^−/−^CD4^+^ T cells was significantly reduced in response to the stimulation of anti-CD3/CD28 Abs (Figure 4F).

### Core Fucosylation Is Essential for TCR–pMHC Conjugates in CD4^+^ T Cell Activation

**Figure 5:**
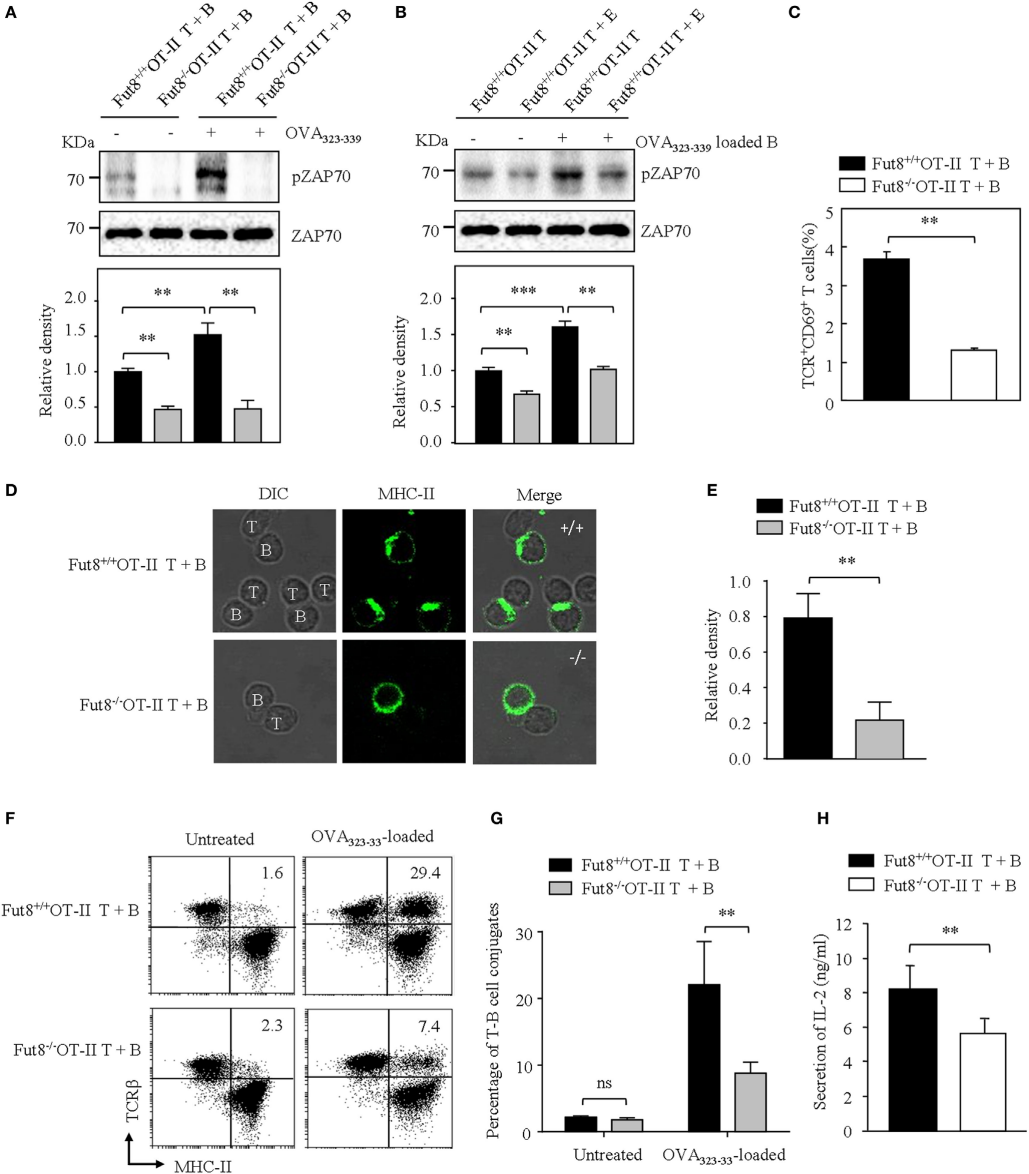

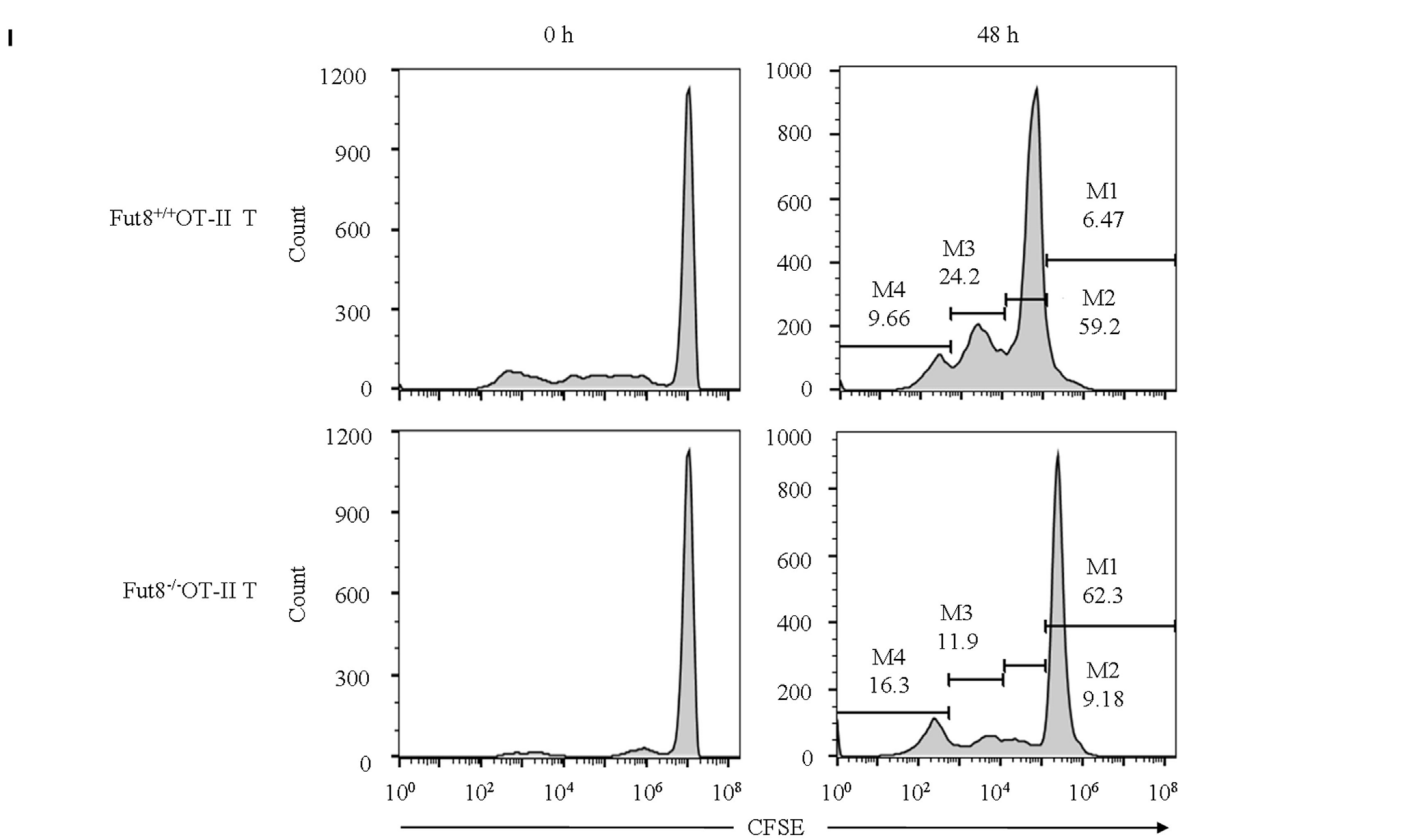
Lack of core fucosulation reduced interaction ability between T cell receptor (TCR) and pMHC-II and impaired the signaling *via* TCR. **(A)** ZAP70 phosphorylation of Fut8^+/+^OT-II T–B cells. (T) B cell conjugate formation was initiated by centrifuging together CD4^+^ T cells with or without 1 µg/mL OVA_323–339_-loaded B cells. The blots were probed by anti-pZAP70 Ab and anti-ZAP70 Ab. Data are representative of three independent experiments. Densitometric analysis of the bands of pZAP70 normalized against ZAP70. Data are shown as the mean ± SD (**p < 0.01). **(B)** ZAP70 phosphorylation of Fut8^+/+^OT-II CD4^+^ T cells with or without fucosidase treatment. Purified CD4^+^ T cells were treated with or without 100 mU Bovine Kidney Fucosidase, and then coincubated with 1 µg/mL OVA_323–339_ loaded Fut8^+/+^OT-II B cells for 30 min. The blots were probed by anti-pZAP70 Ab and anti-ZAP70 Ab. Data are representative of three independent experiments. Densitometric analysis of the bands of pZAP70 normalized against ZAP70. Data are shown as the mean ± SD (**p < 0.01; ***p < 0.001). **(C)** Compare of activation of Fut8^+/+^OT-II T + B cells and Fut8^−/−^OT-II T + B cells. CD4^+^ T cells were conjugated with OVA_323–339_ loaded B cell. After 30 min, the T–B cells were fixed and stained with anti-TCRβ and anti-CD69 Abs. Data are shown as mean values ± SD (**p < 0.01). **(D,E)** Distribution of MHC on the T and B cells. The T–B cells were fixed, permeabilized, and stained with Abs to MHC-II (green). Data are from three separate experiments. The ratio of MHC II intensity at the T–B cell conjugate site relative to non-conjugate areas. Data are shown as the mean values ± SD (**p < 0.01). **(F)** FACS analysis of the conjugates between B cells and CD4^+^ T cells. B cells (OVA_323–339_-loaded or not loaded) were labeled with anti-MHC-II (FITC) and CD4^+^ T cells were labeled with anti-TCRβ (PE-Cy5). T and B cells were quickly mixed and conjugated by a brief centrifugation step. They were then incubated at 37°C for 30 min. A representative staining profile with anti-MHC-II and anti-TCRβ mAb is shown. The conjugate cells are double positive (MHC-II^+^TCRβ^+^) cells. **(G)** Percentage of T–B cell conjugates (both MHC-II and TCRβ positive cells) was calculated. Data are reported as the mean ± SD (**p < 0.01) in four independent experiments. **(H)** The secrition of IL-2 was downregulated in the culture media of Fut8^−/−^OT-II T–B cells. OD values were measured at 492 nm using a microplate reader. Data are reported as the mean ± SD (**p < 0.01) in three independent experiments. **(I)** Proliferation of Fut8^+/+^OT-II CD4^+^ T cells with OVA_323–339_ loaded Fut8^+/+^OT-II B cells. Fut8^+/+^OT-II CD4^+^ T cells were purified and labeled with carboxyfluorescein diacetatesuccinimidyl ester (CFSE). Fut8^+/+^OT-II CD4^+^ T cells were cocultivated with 1 µg/mL OVA_323–339_ loaded Fut8^+/+^OT-II B cells for 48 h, the divided cells were analyzed by FACS. One representative experiment is shown. M1–M4 indicates daughter cell populations which have subsequently lost half of their CFSE signal.

B cells present antigenic peptide with MHC-II molecule as APCs, and CD4^+^ T cells physiologically recognize a complex of a peptide-loaded MHC-II in T–B cell interaction (18). Therefore, we were interested in determining whether core fucosylation is involved in the TCR sensitivity to pMHC-II. To explore the role of core fucosylation on the TCR interaction with pMHC-II ligands, we crossed Fut8^+/−^ mice with OT-II TCR transgenic mice (expressing CD4^+^ TCR specific for OVA_323–339_), and obtained Fut8^+/+^OT-II and Fut8^−/−^OT-II mice. LCA blots analysis showed that the core fucosylation level of T and B cells in Fut8^−/−^OT-II mice (Figure S3 in Supplementary Material). Since MHC-II on the B cell surface can present peptides for recognition and activation of T cells, in this study, B cells were isolated from Fut8^+/+^OT-II and Fut8^−/−^OT-II spleen and incubated with monobiotin-labeled OVA_323–339_. Although electron density was seen for the structures of I-A^d^ covalently linked to an OVA_323–339_, with a single N-glycan (24), the core fucosylation did not affect the peptide presentation abilities of the MHCs between Fut8^+/+^OT-II and Fut8^−/−^OT-II B cells (Figure S4 in Supplementary Material). The CD4^+^ T cells were then stimulated with OVA_323–339_ loaded B cells and subsequent T cell priming was investigated. The freshly purified *ex vivo* CD4^+^ T cells were stimulated with the OVA_323–339_-loaded B cells. As shown in Figure 5A, compared with Fut8^+/+^OT-II CD4^+^ T cells, the phosphorylation level of ZAP-70 was dramatically decreased in Fut8^−/−^OT-II CD4^+^ T cells with stimulation of OVA_323–339_-loaded B cells. Moreover, to remove the cell-surface fucosylation, the Fut8^+/+^OT-II CD4^+^ T cells were treated with 100 mU Glyko^®^ α(1-2,3,4,6) Fucosidase, which can cleaves α1,6-linked fucose more efficiently than other α-fucose linkages (25). The signaling *via* TCR was significantly suppressed in OT-II CD4^+^ T cells treated with this fucosidase, when the T cells were stimulated with OVA_323–339_-loaded B cells (Figure 5B), indicated that the Fut8 inactivation results in the less responsive for TCR stimulation with pMHC-II, despite similar TCR expression levels (Figure 4A). Moreover, the population of TCR^+^CD69^+^ cells was significantly reduced in Fut8^−/−^OT-II CD4^+^ T/OVA_323–339_-loaded B cells compared with Fut8^+/+^OT-II CD4^+^ T/OVA_323–339_-loaded B cells (Figure 5C).

To further determine how Fut8 deficiency affects the T–B cell interactions, the communication of CD4^+^ T cells and B cells was observed by confocal microscopy. OVA_323–339_-loaded MHC-II was markedly increased at the site of T–B cell contact in Fut8^+/+^OT-II T cells for 30 min and was ubiquitous on the Fut8^−/−^OT-II T cell surface (Figures 5D,E). Next, T–B cell conjugates were quantitatively analyzed *via* flow cytometry analysis. Few conjugates of T–B cells were observed in the absence of OVA_323–339_ peptide, whereas peptide-pulsed B cells effectively interacted with CD4^+^ T cells. The percentages of T–B cell conjugates in Fut8^+/+^OT-II and Fut8^−/−^OT-II MHC-II^+^ TCRβ^+^ cells were 29.4 and 7.4%, respectively (Figures 5F,G), indicated that core fucosylation affected the T–B cell interaction. Moreover, the secretion of IL-2 was reduced in the culture media of Fut8^−/−^OT-II T–B cells compared with Fut8^+/+^OT-II T–B cells (Figure 5H). Furthermore, the T cell proliferation with OVA_323–339_-loaded B cells was analyzed by CFSE dilution methods. Compared to the proliferation of Fut8^+/+^OT-II CD4^+^ T cells, those of Fut8^−/−^OT-II CD4^+^ T cells was significantly reduced with the cocultivation of OVA_323–339_-loaded Fut8^+/+^OT-II B cells (Figure 5I). These results indicated that Fut8 deficiency contributes to attenuated T–B cell communication, and follows attenuated T cell activation.

To further elucidate the underlying mechanism of the reduced T–B cell interaction caused by the disruption of Fut8, T and B cell Activation PCR Array was used to compare mRNA expression in Fut8^+/+^ SPLs with that in Fut8^−/−^ SPLs following OVA immunization. As illustrated in Table 1, the expression levels of four genes (CD3e, CD4, CD8, and CD40L) associated with TCR complex formation, eight genes (IL-2Rα, IL-4, IL-6, IL-10, IL-12, IFNγ, CXCL12, and CXCR4) involved in T cell activation, and two genes (CD79a and CD81) associated with B cell activation, were downregulated in Fut8^−/−^ SPLs. Moreover, the gene expressions of signal molecules such as MAPKKK, Vav1, PIK3, PKC, and Cyclin D3 were downregulated in Fut8^−/−^ SPLs. Since core fucosylation of proteins is an important post-translational process, it is not surprising that many molecules involved in T and B cell activation were downregulated in Fut8^−/−^ SPLs.

## Discussion

The glycosylation and Golgi processing pathways have coevolved with the larger regulatory network that controls T cell activation. It is not surprising that changes of glycosylation are linked to AD pathogenesis, such as galactosylation (26, 27) and sialylation (28, 29). The lower levels of sialylated IgG were found in rheumatoid arthritis (RA) and Wegener’s granulomatosis patients, and the sialylated IgG was increased in the sera of patients during remission (28, 29). Moreover, loss of galactose residues on IgG_1_ is showed in the sera of RA patients (26, 27). The significant differences of O-glycan on T helper cells were detected in active SLE patients (30). Core-fucosylated glycans that contain bisecting GlcNAc was increased on the IgG of SLE (31). In this study, core fucosylations were associated with SLE severities, and significantly increased in the CD4^+^ T cells of SLE patients. Coincidently, previous study by Fujii et al. found that the core fucosylation on T cells, required for activation of TCR signaling with anti-CD3/CD28 Abs and induction in colitis, is significantly increased in patients with inflammatory bowel disease (32). Hence, one possible consequence of hyper core fucosylation-induced T cell activation could be the development of SLE. However, the underlying mechanisms of how core fucosylation regulate T cell activation with TCR–pMHC interaction remain unclear.

T cell recognition of pMHC-II ligands on B cells is thought to be carefully coordinated in CD4^+^ T cells. Conformational flexibility is likely responsible for the high degree of promiscuity or cross-reactivity that is evident in the TCR recognition of pMHCs (33–35). There are two models to explain how TCR–pMHC interactions result in T cell activation (36, 37). One model involves an activation threshold based on the occupancy time of the TCR clustering with pMHCs. The alternative model is that pMHC could induce a specific conformational change of TCR complex and influence the quality of signal transductions *via* TCRs. Since the core fucose of N-glycan is located on the cellular surface of T and B cells, and the core fucosylation could affect the flexibility of N-glycan antenna (38) as well as the conformational stability of proteins (39), it is reasonable to assume that core fucosylation of TCR would affect the geometry and conformation of any TCR–pMHC clusters in the T–B cell interactions. Based on a previous study in *D. melanogaster* cells, the closeness of the interaction of Cα with Cβ is shown by the hydrogen bonds of the core fucose residue (6, 17), suggested core fucose possibly strengthens association of Cα with Cβ. A water molecule also bridges the fucose exocyclic oxygen and the side chain of Arg^150β^ (17). In this scenario, core fucosylation is expected to provide exciting opportunities to control the TCR function. In the present study, the percentage of T–B cell conjugates (TCR^+^MHC-II^+^) was reduced by a factor of 3.97 (29.4/7.4%) in Fut8^−/−^OT-II cells, indicated that the core fucosylation has significant functional implications in TCR–pMHC interaction.

Given the intimate relationship between pMHC recognition and TCR signaling, the signaling *via* TCR is also be regulated by glycosylation. It has long been appreciated that Mgat5 deficiency could enhance TCR recruitment to the synapse and results in greater TCR internalization/endocytosis (11). Physiologically, TCR recognition of pMHC-II ligands on APCs such as dendritic cells and B cells are the most important checkpoint for CD4^+^ T cell activation (40). Engagement of the TCR complexes leads to a signaling cascade of protein tyrosine kinases, such as ZAP70. To explore the role of core fucosylation on the T cell activation, we generated Fut8^+/+^OT-II and Fut8^−/−^OT-II mice. Fut8 deficiency results in the attenuated phosphorylation of ZAP70 in Fut8^−/−^OT-II CD4^+^ T cells with OVA_323–339_-loaded B cells. Also, the phosphorylation of ZAP-70 was significantly reduced in Fut8^+/+^OT-II CD4^+^ T cells by the treatment of fucosidase. Moreover, in Fut8^−/−^OT-II cells, the number of CD4^+^ T cells activation (CD69^+^) was decreased by a factor of 3.5. The proliferation of the Fut8^−/−^OT-II CD4^+^ T cells cocultivated with OVA_323–339_-loaded B cells was decreased compared with the Fut8^+/+^OT-II CD4^+^ T cells. It is conceivable that low T–B cell conjugates was proportional to the decreased T cell activation and proliferation in Fut8^−/−^ CD4^+^ T cells. Core fucosylation is likely to be important in all three proposed stages involved in the T cell activation. First, core fucosylation is essential for the TCR structural formation. Second, core fucosylation of TCR could regulate the recognition of pMHC and affect T cell activation threshold. Third, fucose-specific lectins (41) might participate the events in the T–B cell interaction.

Systemic lupus erythematosus is characterized by the overproduction of auto antibodies, mainly IgG. However, B lymphocyte hyperactivity in SLE is T cell dependent. T cells from SLE patients are activated with a decreased activation threshold and regulated abnormally (32). Indeed, overactive CD4^+^ T cells had been implicated in the pathogenesis of SLE (42). Although we know that CD4^+^ T cell deregulation contributes to SLE pathogenesis, but the mechanism is still largely unknown. Studies in SLE patients and murine models of lupus have shown enhanced level of IL-4 (43), IFNγ (44), and IL-6 (45, 46). Compared to Fut8^+/+^ mice, the IgG class-switching was significantly reduced in the sera of Fut8^−/−^ mice after OVA immunization due to low levels of IL-4, IL-5, IL-6, IFNγ, and TGF secreted by Fut8^−/−^CD4^+^ T cells. Development of SLE is associated with genetic, hormonal, environmental, and immunological factors, mainly those related to the helper T cell activation. To investigate the mechanisms of the CD4^+^ T cell activation that is important for SLE pathogenesis, we induced the EAE model using Fut8^+/+^ and Fut8^−/−^ mice. Loss of Fut8 reduced CD4^+^ T cell activation and ameliorated the EAE in Fut8^−/−^ mice. Fut8^−/−^ mice are resistant to the induction of EAE, whereas Mgat5^−/−^ mice are hypersensitive to it (47). In this regard, Fut8 and Mgat5 function as opposing regulators of T cell activation thresholds and susceptibility to AD. The hyper core fucosylations are associated with SLE severities, however, whether altered core fucosylation is a consequence or an underlying cause of the SLE cascade remains unclear.

FUT8 is able to modify multiple proteins, followed by the change of their functions. Because the most of immune response molecules are glycoproteins, FUT8 knockdown/knock out affected their function associated with immune response. Okada et al. (10) showed that loss of core fucosylation caused an inhibitory receptor PD-1 deprivation on the cellular surface and augmented T cell activation, when Fut8^−/−^CD4^+^ T cells transferred into Rag2^−/−^ mice. Although we did not measure the cell-surface PD-1 expression in the T cell activation and T–B cell interaction, the core fucosylation could regulate the PD-1 expression by modulating TCR signaling strength. Physiologically, there may be some equilibria of the core fucosylation on the TCR and PD-1 to regulate T cell activation. The complexity of *in vivo* condition made us cannot conclude the inability for TCR signaling pathway is the sole reason for the T cell activation, but our study first revealed that Fut8 is essential for TCR–pMHC contact and the following CD4^+^ T cell activation. The balance between specific and degenerate T cell recognition of pMHC-II holds important implications for protective immunity versus autoimmunity. With a better understanding of how core fucosylation regulates of the adaptive immune system, its use in therapy for SLE may prove to be a useful intervention.

## Ethics Statement

All animal work was approved by the Ethics Committee at the Dalian Medical University. The Ethics Committee at the hospital approved the study protocol.

## Author Contributions

WL, SM, and SS designed research and performed experiment; ML, ZL, and RY analyzed the experimental data; TM, JG, JZ, and NT corrected paper; WL designed research and wrote paper. All authors reviewed the results and approved the final version of the manuscript.

## Conflict of Interest Statement

The authors declare that the research was conducted in the absence of any commercial or financial relationships that could be construed as a potential conflict of interest.

## Supplementary Material

Video S1 Experimental autoimmune encephalomyelitis symptoms in Fut8^+/+^ mice.

Video S2 Experimental autoimmune encephalomyelitis symptoms in Fut8^−/−^ mice.
