# Supplementary material for: Core Fucosylation of the T Cell Receptor Is Required for T Cell Activation
Source: Front Immunol. 2018 Jan 29;9:78. doi: 10.3389/fimmu.2018.00078 (PMC5796888; doi:10.3389/fimmu.2018.00078)
Supplement: Supplementary file 3 [file Presentation_1.PDF]

Supplementary Material

Core fucosylation of the T cell receptor is required for T cell activation

Wei Liang[1, †], Shanshan Mao[1, †], Shijie Sun[1, †], Ming Li[1], Zhi Li[2], Rui Yu[1], Tonghui Ma[1], Jianguo Gu[3], Jianing Zhang[4], Naoyuki Taniguchi[5] and Wenzhe Li[1, *]

*** Correspondence:** Wenzhe Li: liwenzhe@dlmedu.edu.cn

Supplementary Figures and Tables

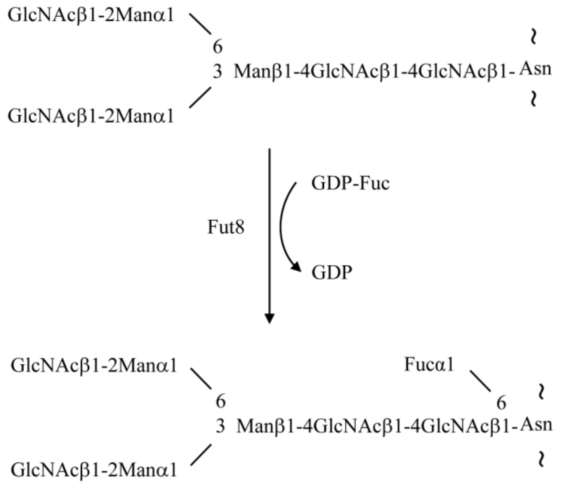

Figure S1. Reaction pathway for the synthesis of core fucose. GDP-fucose is required for the core fucosylation (α1,6 fucosylation) by the Fut8.

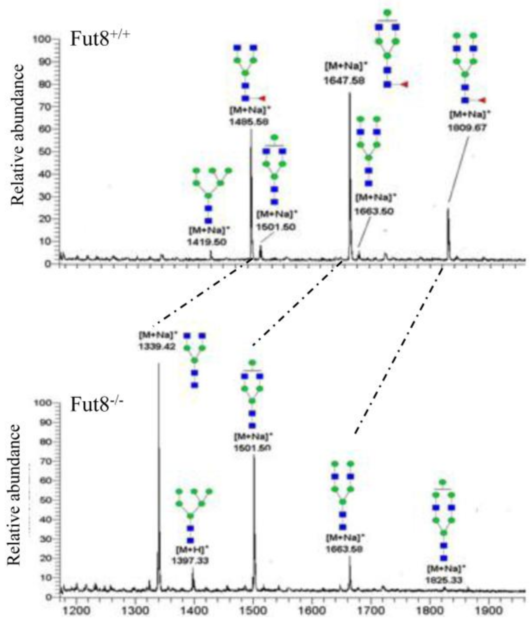

Figure S2. ESI-MS analysis of N-glycans structure of TCRβ from CD4^{+} T cells. Triangle, fucose; square, GlcNAc; circle, mannose. The sample was loaded onto a Sep-Pak C18 solid-phase extraction column. The target N-glycans were eluted with 25% acetonitrile. The samples were injected via a Rheodyne loop with a column of 2 ml and subsequently brought into the electrospray ion source by a stream of 50% methanol (v/v) at a flow rate of 200 ml/min.

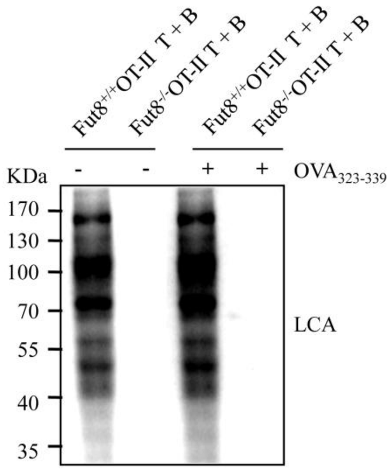

Figure S3. LCA analysis of Fut8$^{+/+}$OT-II T + B cells and Fut8$^{-/-}$OT-II T + B cells. T-B cell conjugate formation was initiated by centrifuging together T cells with or without 1 μg/ml OVA$_{323\text{-}339}$-loaded B cells. Cells were lysated for 15 min at 4 ℃ in lysis buffer and then subjected to 10% SDS-PAGE. The blots were probed by LCA. Data are representative of three independent experiment.

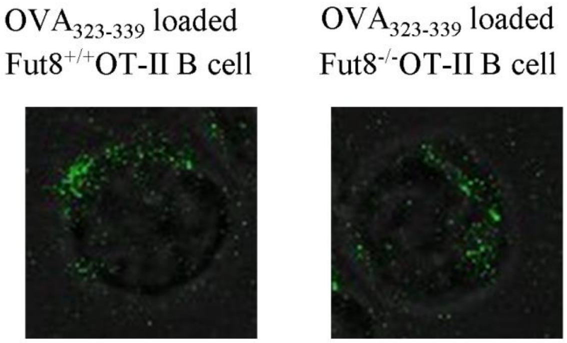

Figure S4. $OVA_{323\text{-}339}$-loading ability of MHC-II on B cells was detected by a confocal microscopy. $Fut8^{+/+}$OT-II cells were purified by MACS sorting. $Fut8^{+/+}$OT-II B and $Fut8^{-/-}$OT-II B cells were incubated with biotin labeled $OVA_{323\text{-}339}$ for 30 min at 37℃ and then stained with avidin-FITC (magnification ×400).

Table S1. Sera samples from the total SLE patients

Number	Sex	ANA titers	Disease
1	Female	1:320	SLE
2	Female	1:320-1:1000	SLE
3	Male	1:320	SLE
4	Female	1:100-1:320	SLE
5	Female	1:320	SLE
6	Male	1:1000	SLE
7	Female	1:320-1:1000	SLE
8	Female	1:1000	SLE
9	Female	1:320	SLE
10	Female	1:100	SLE
11	Female	1:320	SLE
12	Female	1:100	SLE
13	Male	1:320	SLE
14	Female	1:3200	SLE
15	Female	1:3200	SLE
16	Female	1:3200	SLE
17	Female	1:320-1:1000	SLE

SLE patients were investigated by using indirect immune fluorescence assay (IIFA) to detect ANA. The positive rate of ANA is more than 1:100. ANA fluorescence model mainly showed nuclear speckled pattern and nuclear homofeneous pattern. Systemic lupus erythematosus, SLE.

Supplementary Videos

Video S1. EAE symptoms in Fut8$^{+/+}$ mice.

Video S2. EAE symptoms in Fut8$^{-/-}$ mice.
